# Drought-responsive WRKY transcription factor genes *IgWRKY50* and *IgWRKY32* from *Iris germanica* enhance drought resistance in transgenic *Arabidopsis*

**DOI:** 10.3389/fpls.2022.983600

**Published:** 2022-09-06

**Authors:** Jingwei Zhang, Dazhuang Huang, Xiaojie Zhao, Man Zhang, Qian Wang, Xueyan Hou, Dongliu Di, Beibei Su, Shaokun Wang, Pai Sun

**Affiliations:** ^1^College of Landscape Architecture and Tourism, Hebei Agricultural University, Baoding, China; ^2^College of Landscape and Ecological Engineering, Hebei University of Engineering, Handan, China; ^3^State Key Laboratory of North China Crop Improvement and Regulation, Hebei Agricultural University, Baoding, China

**Keywords:** *Iris germanica*, drought tolerance, WRKY transcription factor, stress response mechanisms, *Arabidopsis*

## Abstract

Drought greatly affects the growth and development of garden plants and affects their ornamental value. WRKY transcription factors make up one of the largest transcription factor families in plants and they play an important role in the plant response to drought stress. However, the function of the WRKY gene in response to drought stress in *Iris germanica*, which is commonly used in landscaping, has not been studied. In this study, we isolated two WRKY transcription factor genes from *Iris germanica*, *IgWRKY50* and *IgWRKY32*, which belong to Group II and Group III of the WRKY family, respectively. *IgWRKY50* and *IgWRKY32* could be induced by PEG-6000, high temperature and ABA in *Iris germanica. IgWRKY50* and *IgWRKY32* could quickly respond to drought and they peaked at 3 h after PEG-6000 treatment (19.93- and 23.32-fold). The fusion proteins IgWRKY50-GFP and IgWRKY32-GFP were located in the nucleus of mesophyll protoplasts of *Arabidopsis*. The overexpression of the *IgWRKY50* and *IgWRKY32* genes improved the osmotic tolerance of transgenic *Arabidopsis*, mainly exhibited by the transgenic plants having a higher germination rate and a longer total root length on 1/2 MS medium containing mannitol. Under PEG-6000 stress, the transgenic plants had higher stomatal closure than the wild type (WT). Under natural drought stress, the water loss rate of the isolated leaves of transgenic *Arabidopsis* was lower than that of WT, the contents of proline (Pro) and soluble protein (SP) and the activities of superoxide dismutase (SOD), peroxidase (POD) and catalase (CAT) in the transgenic plants were higher, but the content of malondialdehyde (MDA) was lower. Furthermore, the expression of several stress-related genes (*RD29A, DREB2A, PP2CA*, and *ABA2*) was significantly increased in *IgWRKY50-* and *IgWRKY32*- overexpressing transgenic *Arabidposis* plants after drought treatment. These results suggest that *IgWRKY50* and *IgWRKY32*, as two positive regulators, enhance the drought resistance of transgenic *Arabidopsis* by mediating the ABA signal transduction pathway. *IgWRKY50* and *IgWRKY32* can be used as candidate genes for molecular breeding of drought resistance in *Iris*.

## Introduction

The intensification of the El Niño phenomenon has made the global drought area continue to expand. Drought seriously affects the growth, development and geographical distribution of plants (Anjum et al., 2011). Among the many abiotic stresses, drought damage to plants occupies first place (Shao et al., 2009). Due to the improvement of living standards, people pay more and more attention to ecological environment, and landscape construction has also made substantial developments. However, with the continuous expansion of urban construction, water consumption and maintenance costs of garden green lands have also increased. Water shortage has become one of the important factors restricting ecological construction (Fereres et al., 2003). For garden plants, drought significantly reduces their ornamental value and directly or indirectly affects economic development. Therefore, it is of great significance to thoroughly study the drought resistance mechanism of garden plants and fundamentally solve the contradiction between garden development and water shortages for the construction of energy-saving gardens.

The drought resistance of plants is determined to some extent by the genetic information they carry (Mwadzingeni et al., 2016). When plants are subjected to drought stress, the damage caused by stress is reduced through a series of genetic regulations (Huang et al., 2012). During the process of gene regulation, transcription factors play an important role in responding to stress signals and in controlling gene expression (Singh et al., 2002). Several TF families, such as bZIP, WRKY, NAC, and MYB, are widely involved in the regulation of plant drought stress resistance (Liu et al., 2012; Okay et al., 2014; Rahman et al., 2016; Zhao et al., 2018).

As one of the largest transcription factor families in plants, WRKYs are characterized by the highly conserved WRKY domains that can control gene transcription by specifically binding to the upstream W-box sequence of target genes, and plays an important role in gene regulation (Eulgem et al., 2000). The WRKY domain is a polypeptide sequence composed of 60 highly conserved amino acid residues, which comprises a short-conserved sequence WRKYGQK at the N-terminal and a zinc finger motif at the C-terminal (Eulgem et al., 2000). Although WRKYGQK sequences are highly conserved in plants, some studies have shown that WRKY transcription factors have experienced certain changes during evolution, such as the amino acid mutation of WRKY to WXKY, WSKY, WRRY, WKRY, WKKY, WVKY, or GRKY (Dong et al., 2003; Qiu et al., 2004). In addition to the 7-peptide conserved sequence and zinc finger structure, most WRKY transcription factors also have glutamate-rich regions, proline-rich regions, leucine zipper structures, and serine/threonine-rich regions (Zhang and Wang, 2005). The existence of these structures allows WRKY transcription factors to play multiple roles in gene regulation. According to the number of WRKY domains and the type of the zinc finger structure, WRKY family members are generally divided into three groups. Group I contains two WRKY domains and a C_2_H_2_ (CX_4–5_CX_22–23_HXH) zinc finger. Group II a-e and Group III contain one WRKY domain and a C_2_H_2_ motif or a C_2_HC (CX_7_CX_23_HXC) motif, respectively (Rushton et al., 2010; Yousfi et al., 2016). By analyzing the WRKY family of rice, it was found that the number of Group III gradually increased during the process of evolution, and it was speculated that Group III might be more active in evolution than Group I and Group II (Wu et al., 2005).

WRKY transcription factors are indispensable response factors for plants to resist biotic and abiotic stresses (Jiang et al., 2017). Overexpression of the WRKY transcription factor genes in plants can improve the resistance of plants to adversity. *GsWRKY20* in wild soybean was overexpressed in *Arabidopsis*, and the transgenic lines showed stronger tolerance to drought stress (Luo et al., 2013). The *OsWRKY45* cloned from rice was overexpressed in *Arabidopsis*, which enhanced the disease resistance and drought tolerance of *Arabidopsis*, indicating that *OsWRKY45* is involved in biotic and abiotic stress response signal transduction (Qiu and Yu, 2009). The *ZmWRKY58* gene isolated from maize was overexpressed in rice, which enhanced the tolerance of the transgenic rice to drought and salt stress (Cai et al., 2014). At present, research on the function of WRKY transcription factors is related to high- (Cheng et al., 2021) and low-temperature resistance (Romero et al., 2019), high-salt resistance (Li et al., 2013), drought resistance (He et al., 2016), heavy metal resistance (Wu et al., 2022) and pest resistance (Xiong et al., 2019). However, these studies mainly focused on model plants such as *Arabidopsis* (Dong et al., 2003), rice (Yu et al., 2010), and tobacco (Maeo et al., 2001), as well as crops such as maize (Wang et al., 2018), barley (Mangelsen et al., 2008), and cotton (Dou et al., 2014). There are few studies on the regulatory mechanism of WRKY transcription factors in garden plants under stress.

*Iris germanica* is a perennial herbaceous flower with good ornamental characteristics, and it is drought-resistant, water-saving and barren-resistant. It is the species with the highest ornamental value among irises and is widely used in landscaping (Zhang and Chen, 2013). The drought resistance of *Iris germanica* is obviously higher than that of other plants of the same genus (Han et al., 2006). *Iris germanica* is not only an important greening material for energy-saving garden construction but also a genetic treasure trove for drought-resistant breeding of *Iris* and other garden plants. It is of great value to deeply study the drought resistance of *Iris germanica*.

At present, the research of *Iris germanica* mainly focuses on the determination of physiological and biochemical indicators under abiotic stress (Bo et al., 2017; Zhao et al., 2021), hybrid breeding (Azimi and Alavijeh, 2021), phenotype analysis (Fan et al., 2022), and exploration of flowering mechanism (Fan et al., 2020). There are few studies on stress-resistant genes, and they are limited to the detection of gene expression (Wang et al., 2021), and the function of gene has not been verified. The mechanism of WRKY gene under abiotic stress in *Iris germanica* has not been studied. In our previous work, we completed the transcriptome sequencing of “Little Dream,” a high drought-resistant cultivar of *Iris germanica*, and identified 20 differentially expressed genes belonging to the WRKY transcription factor family (Zhang et al., 2021). To further verify the role of WRKY transcription factors in stress resistance regulation, two WRKY transcription factor family genes, *IgWRKY50* and *IgWRKY32*, belonging to WRKY transcription factor family Group II and Group III, respectively, were isolated from “Little Dream.” The drought resistance function of the *IgWRKY50* and *IgWRKY32* genes was verified in transgenic *Arabidopsis*. The results showed that the overexpression of these two genes improved the drought resistance of transgenic *Arabidopsis*. This result fills the gap in the study of abiotic stress resistance of the *Iris germanica* WRKY gene, provides a key clue for understanding the role of the WRKY gene in the drought response of *Iris germanica*, and provides excellent candidate genes for drought resistance breeding of *Iris* and other garden plants.

## Materials and methods

### Stress treatment of *Iris germanica*

The 1-year-old *Iris germanica* cultivar “Little Dream” was used as the experimental material. Seedlings at the vegetative growth stage that exhibited strong and similar growth were selected and cultured in 1/2 Hongland solution for 7 days. Plant culture was carried out in an artificial climate chamber with a temperature of 25°C, a photoperiod of 16 h/8 h and a light intensity of 180 μmol m^–2^s^–1^. Then, the plants were treated with 1/2 Hoagland solution containing 200 g⋅L^–1^ PEG 6000, 150 mM NaCl and 100 μM ABA for 24 h in the same chamber. Low- and high-temperature treatments were conducted in 4 and 42°C chambers for 24 h, respectively. All abiotic stresses were initiated on March 15, 2021. The seedlings cultivated in regular 1/2 Hongland solution were used as a control. For the multiple stress experiments, samples were taken at 0, 1, 3, 6, 12, and 24 h after PEG 6000, NaCl, ABA, low- and high-temperature treatments. The samples were collected from the middle and upper parts of one to two mature leaves next to the center leaf. For different organ expression pattern analyses, the leaves, fibrous roots, tubers, flag petals, and fall petals of the control seedlings were collected at 0 h. All samples were wrapped in tin foil, immediately frozen in liquid nitrogen and stored at –80°C until RNA extraction. The 1/2 Hongland solution used in the experiment was refreshed every day. Leaves of intact plants were always selected as material for the next experiments. Each treatment was repeated three times. The specific gene primers are listed in Supplementary Table 1A.

### Gene isolation and bioinformatics analysis of *IgWRKY50* and *IgWRKY32*

Two groups of specific primers (Supplementary Table 1B) were designed with Primer Premier 5.0 software (Premier, Canada), and the complete coding sequences of the *IgWRKY50* and *IgWRKY32* genes were amplified twice. The PCR products were connected to the pEASYT3 cloning vector (TransGen Biotech, Beijing, China) and then sequenced by the company (Sangon Biotech, Shanghai, China). The open reading frames of *IgWRKY50* and *IgWRKY32* were found by NCBI ORF Finder.^1^ The protein functional domains of *IgWRKY50* and *IgWRKY32* were predicted by SMART software (Letunic and Bork, 2018; Letunic et al., 2021).^2^ The phylogenetic tree of amino acid sequences of other species with homology to *IgWRKY50* and *IgWRKY32* genes was constructed by MEGA 6 software using the neighbor-joining (NJ) method with 1,000 bootstrap replicates (Tamura et al., 2013). The accession numbers of the WRKYs are listed in Supplementary Table 2. The tertiary structure prediction of *IgWRKY50* and *IgWRKY32* proteins was completed by SWISS-MODEL software (Biasini et al., 2014), and the three-dimensional structure model of proteins was constructed by the I-TASSER website^3^ and Chimera 1.11.2 software^4^ (Pettersen et al., 2004).

### Subcellular localization analysis of *IgWRKY50* and *IgWRKY32*

The expression vector PBI221-GFP (YRGene, Changsha, China) was used for an investigation of subcellular localization. The coding regions of *IgWRKY50* and *IgWRKY32* were fused to the PBI221-GFP vector containing the CaMV35S promoter. The specific primers are listed in Supplementary Table 3A. *Arabidopsis* protoplasts were isolated from 3-week-old leaves of *Arabidopsis* (Columbia-0). The recombinant plasmids PBI221-IgWRKY50-GFP and PBI221-IgWRKY32-GFP and the nuclear localization plasmid pHBT-NLS-mCherry were transferred into *Arabidopsis* protoplasts by the PEG-mediated method (Sheen, 2001) and incubated at 22°C for 16 h in the dark. The empty vector was used as a control. The GFP fluorescence signals were observed by a laser confocal microscope (Olympus FV10-ASW, Tokyo, Japan).

### Plant transformation and generation of overexpressing *Arabidopsis* plants

The complete coding sequences of *IgWRKY50* and *IgWRKY32* were ligated into the binary vector PBI121 (YRGene, Changsha, China) containing Xbal and Smal restriction sites under the control of the *Cauliflower mosaic virus* 35S (CaMV35S) promoter using specific primers (Supplementary Table 3B). The recombinant plasmid was introduced into *Agrobacterium tumefaciens* strain *GV3101* (AngYuBio, Shanghai, China) by the freeze–thaw method. A transgenic *Arabidopsis* strain was obtained by transforming wild-type (WT) *Arabidopsis* (Columbia-0) by the floral dip method (Clough and Bent, 2010). T1 plants were screened on 1/2 MS medium containing 0.1% kanamycin. T1 seedlings were confirmed by PCR and selfed through two more generations to generate T3 transgenic progeny. T3 seedlings confirmed by sequencing (Sangon Biotech, Shanghai, China). Twenty-five *IgWRKY50* overexpression lines and forty-four *IgWRKY32* overexpression lines were obtained. Three lines with higher expression levels were selected from the *IgWRKY50* and *IgWRKY32* transgenic lines by RT–qPCR for further analysis. The method used to clean the *Arabidopsis* seeds was described by Feng et al. (2015).

### Determination of water loss rate

WT and transgenic *Arabidopsis* plants growing normally for 21 days in substrate (V_nutrient soil_: V_vermiculite_ = 1:1) were used to calculate the water loss rate of the isolated leaves. WT and transgenic *Arabidopsis* lines with basically the same growth were selected. Five rosette leaves of a similar size were taken from each line, and the leaves were placed face up in a petri dish at room temperature. Losses in fresh weight were monitored at 0.5, 1, 2, 4, 6, 8, and 12 h. Water loss is expressed as the percentage of initial fresh weight. Three replicates were performed. All types of *Arabidopsis* were cultured in an artificial climate room. The culture conditions were a constant temperature at 22°C, photoperiod of 16 h/8 h, light intensity of 150 μmol m^–2^s^–1^ and relative humidity of 70%.

### Measurement of antioxidant enzymes, malondialdehyde, proline and soluble protein

WT and transgenic *Arabidopsis thaliana* plants with normal growth for 21 days were adequately irrigated before inducing a drought, which lasted for 14 days. The physiological parameters of the leaves were measured at 14 d after treatment. All types of *Arabidopsis* grown under normal water conditions were used as controls. The activities of superoxide dismutase (SOD), catalase (CAT), and peroxidase (POD) were determined by the corresponding kits (Nanjing Jiancheng Bioengineering Institute, China). The MDA content was measured using a thiobarbituric acid (TBA) reaction (Zhang et al., 2021). The accumulation of Pro in the leaves was measured using the acid ninhydrin method (Fu et al., 2016). The SP content was determined by Coomassie Brilliant Blue G-250 staining (Fu et al., 2016).

### Stress tolerance assay in overexpressing *Arabidopsis* plants

To determine the seed germination rate of various types of *Arabidopsis*, sterilized seeds of WT and transgenic *Arabidopsis* were spread on 1/2 MS medium containing 0, 100, 150, and 200 mM mannitol and cultured in an *Arabidopsis* artificial climate chamber for 7 days. The seed germination rate was recorded every day. Thirty-six seeds were taken from each strain, and each experiment was repeated 3 times. The seeds were vernalized at 4°C for 3 days in advance. Seeds were considered to be germinated when radicles emerged from the seed coats.

To compare the total root growth of various types of *Arabidopsis*, the sterilized seeds of WT and transgenic *Arabidopsis* strains were cultured in 1/2 MS medium for 3 days and then transferred to 1/2 MS medium containing 0 mM and 150 mM mannitol for 7 days. Then, the total root lengths of various types of *Arabidopsis* were measured and recorded by photographs. Thirty seeds were taken from each strain, and each experiment was repeated 3 times.

To observe the phenotypic changes of various types of *Arabidopsis thaliana* under natural drought, the sterilized seeds of the WT and transgenic lines were sown on 1/2 MS medium and transplanted to the substrate (V_nutrient soil_:V_vermiculite_ = 1:1) after 10 days. Eighteen plants were selected from each line. The phenotype was photographed after normal culture in an *Arabidopsis* artificial climate culture room for 3 weeks. After that, the irrigation was stopped, and the phenotype was photographed again after 21 days of drought.

To observe the stomatal opening and closing of various types of *Arabidopsis thaliana*, the rosette leaves of WT and transgenic *Arabidopsis* lines grown for 21 days were used as materials. The leaves were floated in stomata buffer containing 50 μM CaCl, 10 mM KCl, and 10 mM Mes-Tris, pH = 6.15, under the light of 80 μmol m^–2^s^–1^ for 2 h until the stomata were fully opened (Akter et al., 2012). After that, the leaves were transferred into stomatal buffer containing 8% PEG-6000, and stomatal opening and closing were observed every 15 min. When the stomatal opening and closing changed, the lower epidermis of the leaves was torn off to make temporary mounts, and the stomatal images were collected with an optical microscope (Olympus FV10-ASW, Tokyo, Japan). Fifteen stomatal apertures were measured for each sample. The length and width of the stomata were determined by ImageJ software,^5^ which was used to calculate the stomatal aspect ratio.

### Expression profile of stress-related genes

To elucidate the possible molecular mechanisms of *IgWRKY50* and *IgWRKY32*, the expression levels of *RD29A, DREB2A, PP2CA*, and *ABA2* were assessed in transgenic and WT plants after 14 days of continuous drought by RT–qPCR. The specific primers are listed in Supplementary Table 3C.

### RNA extraction and qRT–PCR analysis

Total RNA was isolated using TRIzol reagent (Life Technologies, Inc., Grand Island, NY, United States) following the manufacturer’s protocol. A 1 μg RNA sample was reverse transcribed using a HiFiScript cDNA Synthesis Kit (CWBIO, Beijing, China). Gene-specific primers were designed using Primer Premier 5.0 (Premier, Canada) software. RT–qPCR was performed with Fast Super EvaGreen qPCR Master Mix (US Everbright Inc., Jiangsu, China) on an ABI Prism 7500 system (Applied Biosystems, Waltham, MA, United States). The PCR volume was 20 μL, which included 10 μL of 2 × Fast Super Eva Green Master Mix, 0.5 μL of the forward primer, 0.5 μL of the reverse primer, 0.2 μL of 10 × ROX, 2 μL of the cDNA template, and 6.8 μL of denucleated acid water. The reaction conditions were 2 min at 95°C for denaturation followed by 45 cycles of 5 s at 95°C, 5 s at 60°C, and 50 s at 72°C. The RT–qPCR analysis consisted of three biological and three technical replications. The relative expression levels of the genes were calculated using the 2^–ΔΔ^*^Ct^* method.

### Statistical analysis

The data were analyzed using Microsoft Office Excel 2010 software and IBM SPSS 21.0 (IBM Corp., NY, United States), and Duncan’s multitest and Student’s *t*-test were used to evaluate the significance of the differences. GraphPad Prism 7.0 was used to generate the charts.

## Results

### Sequence analysis of *IgWRKY50* and I*gWRKY32*

**FIGURE 1:**
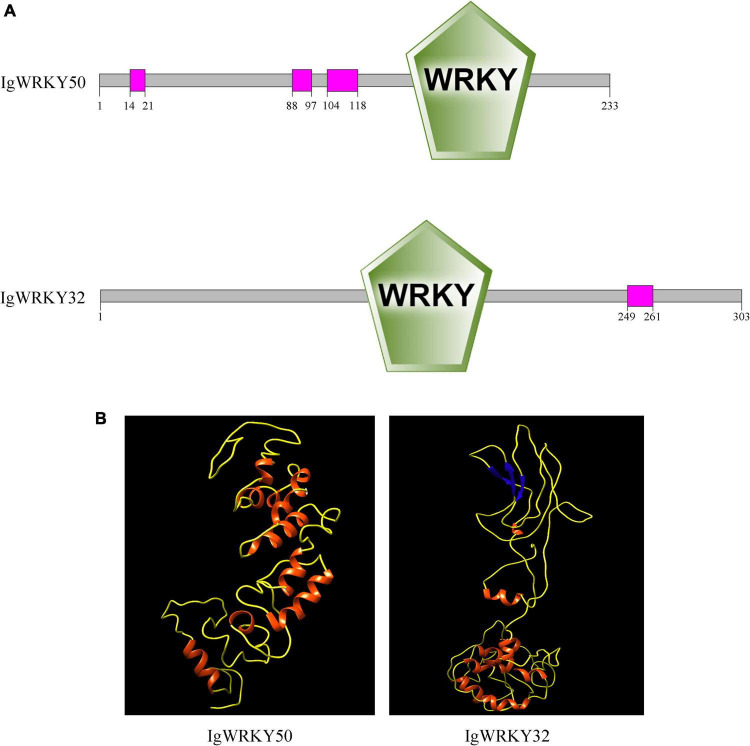
Protein domain organization **(A)** and three-dimensional structures **(B)** of *IgWRKY50* and *IgWRKY32.*

In this study, *IgWRKY50* (GenBank Accession No. ON571660) and *IgWRKY32* (GenBank Accession No. ON571661) were cloned and isolated from *Iris germanica* “Little Dream.” The complete coding sequence of *IgWRKY50* was 702 bp, encoding 233 amino acids. The unique WRKYGKK sequence of the WRKY transcription factor family was found at amino acid sequence 146, and the zinc finger structure was the C2H2 type. The complete coding sequence of *IgWRKY32* was 912 bp, encoding 303 amino acids. The unique WRKYGQK sequence of the WRKY transcription factor family was found at amino acid sequence 129, and the zinc finger structure was the C2HC type (Supplementary Figures 1A,B). Using SMART software to analyze the gene structure, it was found that the proteins encoded by *IgWRKY50* and *IgWRKY32* had typical domains of the WRKY family. *IgWRKY50* was included in three low-complexity regions located at amino acids 14–21, 88–97, and 104–118. *IgWRKY32* was included in one low-complexity region located at amino acids 249–261 (Figure 1A).

**FIGURE 2:**
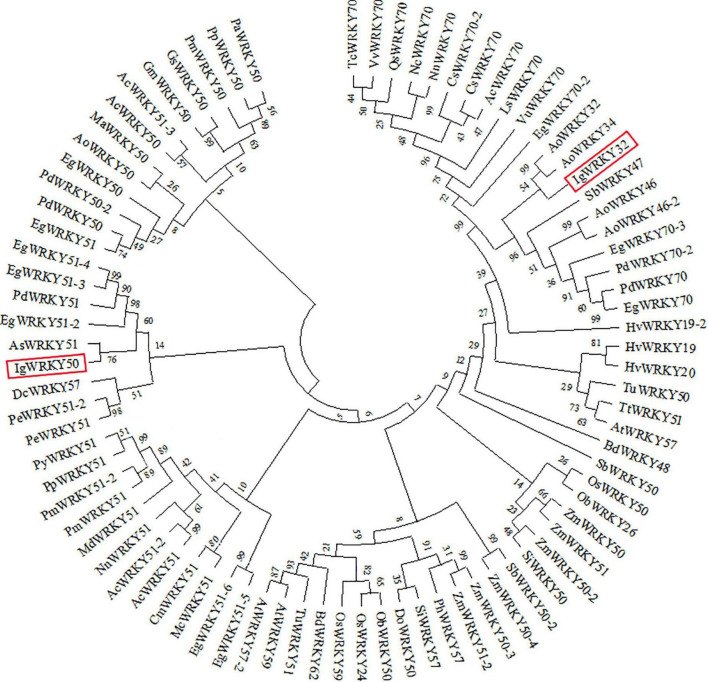
Phylogenetic relationship of *IgWRKY50* and *IgWRKY32* with their orthologous in other plant species. The phylogenetic tree was based on comparisons of amino acid sequences and produced by MEGA 6.0 software.

The amino acid sequences of *IgWRKY50* and *IgWRKY32* were compared by BLAST, and it was found that the homology between the *IgWRKY50* and *MaWRKY50* proteins of *Musa acuminata* subsp. *malaccensis* was the highest, at 82.56%. *IgWRKY32* had the highest homology with the *AoWRKY32* protein of *Asparagus officinalis*, which was 76.62%, so the two genes were named *IgWRKY50* and *IgWRKY32*, respectively (Supplementary Tables 4A,B). *IgWRKY50* and *IgWRKY32* had similar tertiary structures, and both belonged to DNA-binding proteins, which were mainly composed of uncoiled structures and α-helices (Figure 1B). The crystal structures of *IgWRKY50* and *IgWRKY32* were highly similar to the conserved domains of the rice stress-responsive transcription factor *OsWRKY45*, and similar amino acids accounted for 43.55 and 46.88% of the total amino acid residues, respectively. Phylogenetic trees of 81 WRKY transcription factor family genes with high similarity to the *IgWRKY50* and *IgWRKY32* genes in 39 species were constructed by MEGA 6.0 software (Supplementary Table 2). The results showed that *IgWRKY50* was closely related to *AsWRKY51* of *Apostasia shenzhenica*, and *IgWRKY32* belonged to the same branch as *AoWRKY32* and *AoWRKY34* of *Asparagus officinalis* (Figure 2). It was speculated that *Iris germanica*, *Apostasia shenzhenica* and *Asparagus officinalis* belonged to Liliidae, so they had high homology.

### Transcription profiles of *IgWRKY50* and *IgWRKY32* under abiotic stress

**FIGURE 3:**
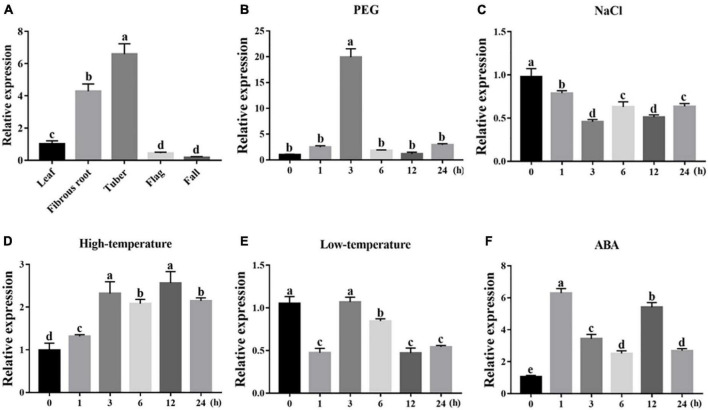
Transcription profiles of *IgWRKY50*. **(A)** Organ expression assay of *IgWRKY50* in different *Iris germanica* organs (leaf, fibrous, tuber, flag, fall). Transcription profiles of *IgWRKY50* under 200 g⋅L^–1^ PEG6000 **(B)**; 150 mM NaCl **(C)**; 42°C **(D)**; 4°C **(E)**; 100 μM ABA **(F)** treatments in *Iris germanica* leaves. The transcriptional level at time point 0 h (for the multiple stress experiments) and the leaf (for the organ expression assay) was defined as 1.0.

**FIGURE 4:**
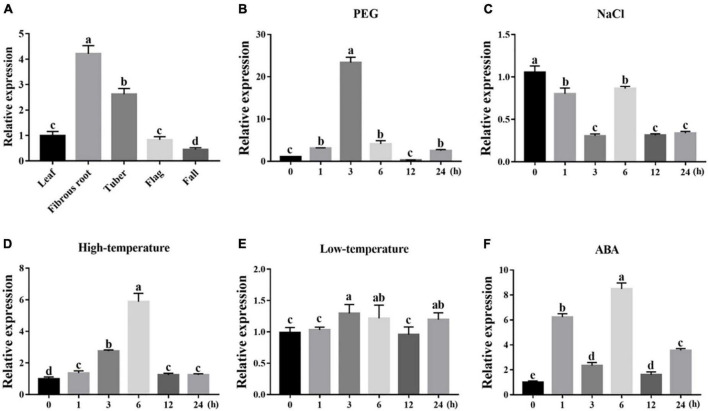
Transcription profiles of *IgWRKY32*. **(A)** Organ expression assay of *IgWRKY32* in different *Iris germanica* organs (leaf, fibrous, tuber, flag, fall). Transcription profiles of IgWRKY32 under 200 g⋅L^–1^ PEG6000 **(B)**; 150 mM NaCl **(C)**; 42°C **(D)**; 4°C **(E)**; 100 μM ABA **(F)** treatments in *Iris germanica* leaves. The transcriptional level at time point 0 h (for the multiple stress experiments) and the leaf (for the organ expression assay) was defined as 1.0. The vertical ordinates represent fold changes and the horizontal ordinates represent treatment times. Error bar represent standard deviations (*SD*). The data represent means ± *SD* of three biological replications. Different letters in bar graphs indicate significant differences at *p* < 0.05. ABA-abscisic acid.

To dissect their potential functions, the expression of *IgWRKY50* and I*gWRKY32* was investigated in different tissues and under various stress conditions by RT–qPCR. The results showed that *IgWRKY50* and *IgWRKY32* were expressed in all organs, *IgWRKY50* had the highest expression in tubers, and *IgWRKY32* had the highest expression in fibrous roots, indicating that *IgWRKY50* and *IgWRKY32* had important functions in tubers and fibrous roots of *Iris germanica*, respectively (Figures 3A, 4A). Transcription of *IgWRKY50* and *IgWRKY32* was induced by PEG-6000, high temperature and ABA treatments, while it was not affected by NaCl and low temperature. The transcriptional levels of *IgWRKY50* and *IgWRKY32* peaked at 3 h after PEG-6000 treatment and were 19.93- and 23.32-fold higher than those of the control, respectively. Under high-temperature treatment, the transcriptional level of *IgWRKY50* was upregulated continuously and it reached its peak at 12 h (2.56-fold). The transcriptional level of *IgWRKY32* increased at first, reached its peak at 6 h (5.88-fold), and then it decreased rapidly to a level similar to that of the control. Under ABA treatment, the transcriptional levels of *IgWRKY50* and *IgWRKY32* peaked at 1 h (6.30-fold) and 6 h (8.49-fold), respectively (Figures 3B–F, 4B–F).

### *IgWRKY50* and *IgWRKY32* were localized in the nucleus

**FIGURE 5:**
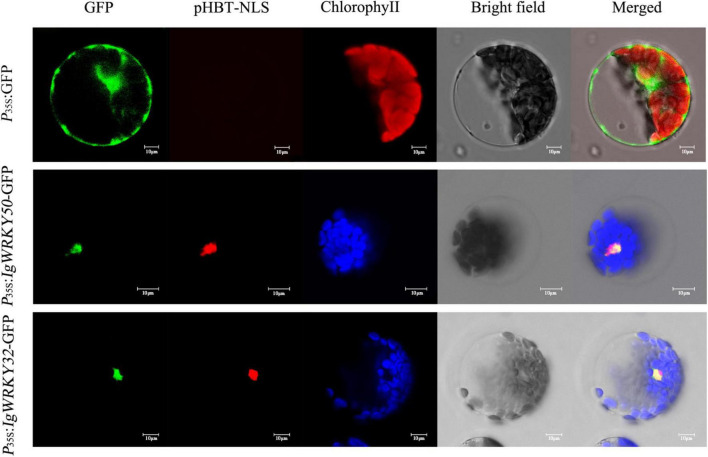
Subcellular localization of the *IgWRKY50* and *IgWRKY32*. P_35S_:IgWRKY50-GFP, P_35S_:IgWRKY32-GFP, and P_35S_: GFP control vectors were transiently expressed in *Arabidopsis* leaf protoplasts. Scale bar = 10 μm.

The complete coding regions of *IgWRKY50* and *IgWRKY32* were cloned into the PBI221-GFP vector under the control of the CaMV 35S promoter. The protoplasts of *Arabidopsis* leaves were transformed and transferred into the nuclear targeting plasmid pHBT-NLS-mCherry. The empty vector was used as a control. The GFP signals of 35S:IgWRKY50-GFP and 35S:IgWRKY32-GFP were exclusively observed in the nucleus and they overlapped with the red fluorescence emitted by the Maker-localized NLS protein in the nucleus, whereas the GFP signal of the control was discovered in the cytoplasm and nucleus (Figure 5). Therefore, *IgWRKY50* and *IgWRKY32* likely function as transcription factors in the nucleus.

### Overexpression of *IgWRKY50* and *IgWRKY32* enhanced drought tolerance in transgenic plants

**FIGURE 6:**
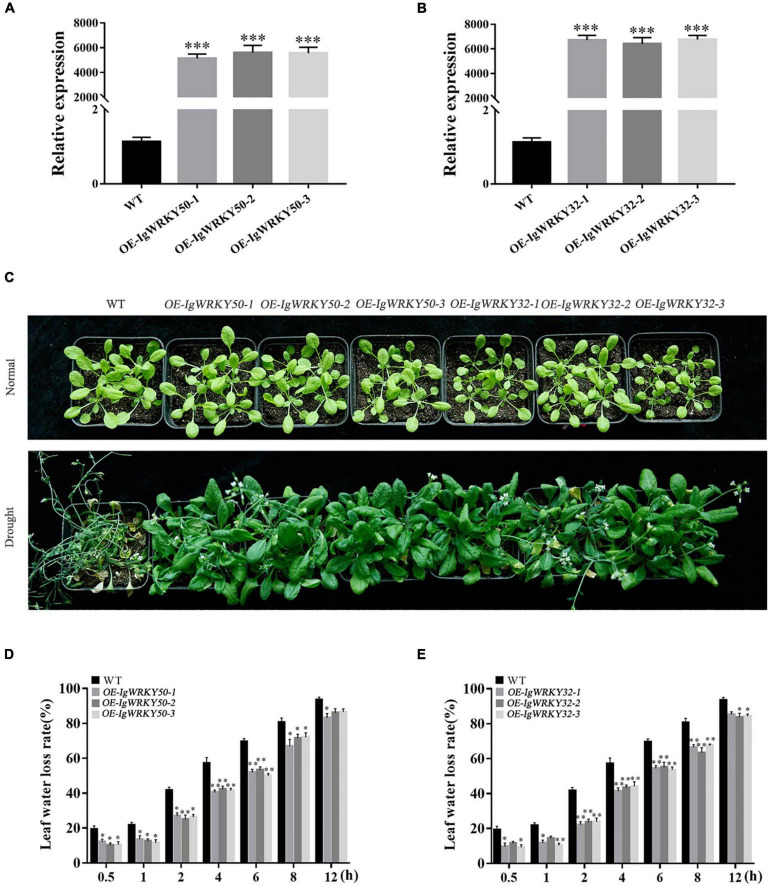
Phenotype analysis and tolerance assay of transgenic *Arabidopsis* and WT under drought treatment. Gene validation of *IgWRKY50* **(A)** and *IgWRKY32* **(B)** in transgenic *Arabidopsis* lines by qRT-PCR. Phenotype analysis of WT and the transgenic *Arabidopsis* under drought treatment **(C)**. The water loss rate of WT and *IgWRKY50* transgenic plants under drought condition **(D)**. The water loss rate of WT and *IgWRKY32* transgenic plants under drought condition **(E)**. Data are means ± *SD* of three independent experiments, and asterisks (*or**or***) represent the significant differences at *p* < 0.05 or *p* < 0.01 or *p* < 0.001, respectively (Student’s *t*-test).

*IgWRKY50* and *IgWRKY32* under the control of CaMV35S were transformed into *Arabidopsis*. Three highly expressed lines (OE-*IgWRKY50*-1, OE-*IgWRKY50*-2, OE-*IgWRKY50*-3, and OE-*IgWRKY32*-1, OE-*IgWRKY32*-2, OE-*IgWRKY32*-3) were selected from T3 homozygous lines with *IgWRKY50* and *IgWRKY32* to study their characteristics. The qRT-PCR analysis showed that *IgWRKY50* and *IgWRKY32* were detected in transgenic *Arabidopsis* plants, but not in WT (Figures 6A,B). The water loss rates of WT, *IgWRKY50*-overexpressing and *IgWRKY32*-overexpressing *Arabidopsis* lines were evaluated within a specified time interval. The results showed that the leaf water loss rate of the *IgWRKY50*- and *IgWRKY32*-overexpressing lines was lower than that of the WT, but there was no significant difference between the *IgWRKY50*- and *IgWRKY32*-overexpressing lines (Figures 6D,E). The 3-week-old WT and transgenic *Arabidopsis* seedlings were exposed to drought conditions for 21 days, and it was found that all types of *Arabidopsis* grown for 3 weeks under normal conditions grew robustly and had basically the same growth. After drought treatment, all of the leaves of the WT were seriously curled and withered and yellowed, while a few leaves of the *IgWRKY50*- and *IgWRKY32*-overexpressing lines showed curling and yellowing at the tip. Compared with the other transgenic lines, OE-*IgWRKY50*-1 and OE-*IgWRKY32*-2 had a relatively higher degree of leaf wilting, but their overall growth was good, and the yellowing and dryness degree of the leaves was significantly lower than that of the WT (Figure 6C).

**FIGURE 7:**
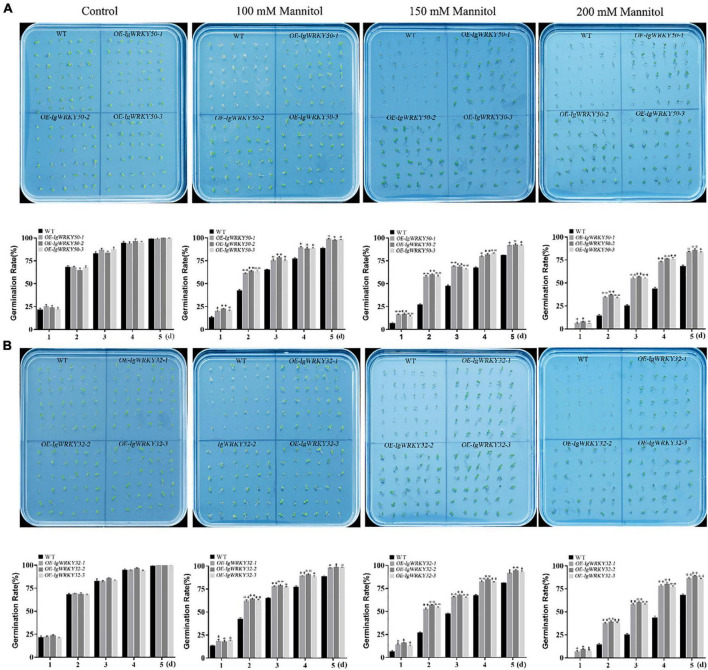
Germination of WT and transgenic *Arabidopsis* lines under mock drought stress. Seed germinations of WT and *IgWRKY50* transgenic *Arabidopsis* lines on ½ MS medium with 0 Mm, 100 mM, 150 mM, 200 mM Mannitol **(A)**. Seed germinations of WT and *IgWRKY32* transgenic *Arabidopsis* lines on ½ MS medium with 0 mM, 100 mM, 150 mM, 200 mM Mannitol **(B)**. Data are means ± *SD* of three independent experiments, and asterisks (*or**) represent the significant differences at *p* < 0.05 or *p* < 0.01, respectively (Student’s *t*-test).

To assess the osmotic stress tolerance of the transgenic *Arabidopsis* plants, WT and *IgWRKY50*- and *IgWRKY32*-overexpressing *Arabidopsis* seeds were sown on 1/2 MS medium containing mannitol (0, 100, 150, and 300 mM), and the seed germination rate was monitored for 5 days. There was no significant difference in seed germination between the transgenic lines and WT on the same medium without mannitol. On the medium containing 100 mM mannitol, the *IgWRKY50* and *IgWRKY32* transgenic seed germination rates were 97.62 and 98.08%, respectively, and the seed germination rate of WT was 88.28%. On the medium containing 150 mM mannitol, the seed germination rate of WT was 80.49%, while that of *IgWRKY50* and *IgWRKY32* was more than 90%. On the medium containing 200 mM mannitol, the transgenic seed germination rates of *IgWRKY50* and *IgWRKY32* were 83.81 and 86.56%, respectively, and the seed germination rate of WT was only 67.90% (Figure 7). The results indicated that the overexpression of *IgWRKY50* and *IgWRKY32* genes improved the osmotic stress tolerance of transgenic *Arabidopsis* seeds during germination.

**FIGURE 8:**
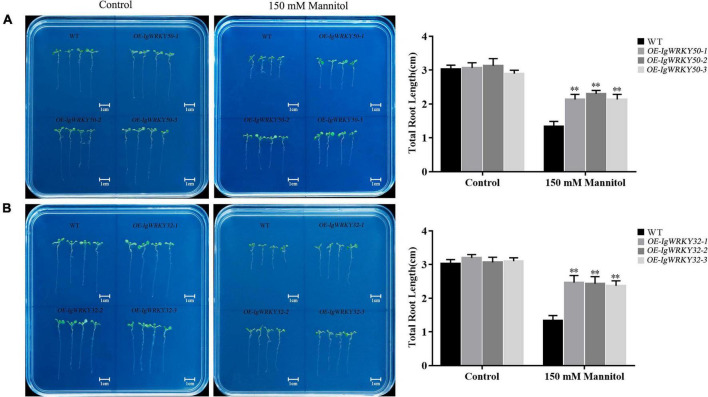
Root elongation of WT and transgenic *Arabidopsis* lines under mock drought stress. Root elongation of WT and *IgWRKY50* transgenic *Arabidopsis* lines on 1/2 MS medium with 0 and 150 mM Mannitol **(A)**. Root elongation of WT and *IgWRKY32* transgenic *Arabidopsis* lines on ½ MS medium with 0 and 150 mM Mannitol **(B)**. Scale bar = 1 cm. Data are means ± *SD* of three independent experiments, and asterisks (**) represent the significant differences at *p* < 0.01 (Student’s *t*-test).

The transgenic lines and WT seeds were cultured on 1/2 MS medium at 22°C for 3 days and then transferred to 1/2 MS medium containing 0 and 150 mM mannitol for 7 days. On the medium without mannitol, the total roots of the transgenic lines and WT seedlings grew normally, and their lengths were basically the same. On the medium containing 150 mM mannitol, the growth of the total roots of the WT and transgenic lines was inhibited, and the inhibition degree of the WT was more obvious than that of the transgenic lines. The length of the total roots of the *IgWRKY32* strains was slightly longer than that of the *IgWRKY50* strains, but there was no significant difference (Figure 8). These results further confirmed that the overexpression of *IgWRKY50* and *IgWRKY32* genes enhanced the drought tolerance of the transgenic *Arabidopsis* plants.

**FIGURE 9:**
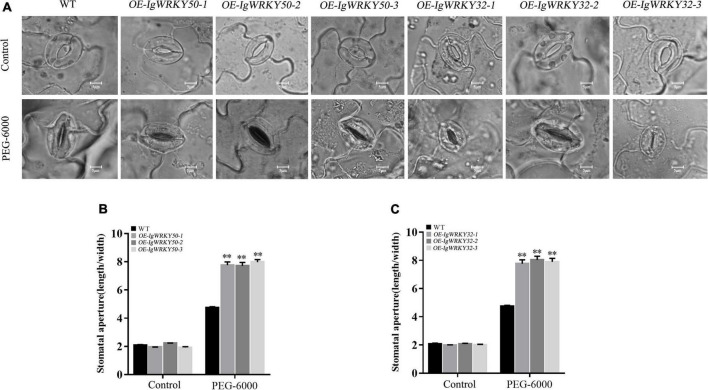
Stomatal opening and closing of WT and transgenic *Arabidopsis* leaves at seeding stage under 8% PEG-6000 stress. Stomatal closure of leaves of WT and transgenic lines **(A)**. Measurements of stomatal apertures (width/length) of *IgWRKY50* transgenic lines and WT plants **(B)**. Measurements of stomatal apertures (width/length) of *IgWRKY32* transgenic lines and WT plants **(C)**. Scale bar = 5 μm. Data are means ± *SD* of three independent experiments, and asterisks (**) represent the significant differences at *p* < 0.01 (Student’s *t*-test).

In addition, we also observed stomatal opening and closing in leaves of transgenic lines and WT under osmotic stress. There was no significant difference in the degree of stomatal opening of each type of *Arabidopsis* before PEG-6000 treatment, while the degree of stomatal closure of the transgenic lines after treatment was more compact than that of the WT (Figure 9A). The ratio of the stomatal length to width also showed that the stomatal closure degree of the transgenic lines was stronger than that of the WT, but there was no significant difference among the transgenic lines (Figures 9B,C). Based on this result, it was speculated that the transgenic *Arabidopsis* could quickly close the stomata and reduce its transpiration rate to retain water, thereby obtaining a strong ability to resist drought stress in arid environments.

### Overexpression of *IgWRKY50* and *IgWRKY32* changes the physiological and biochemical levels of the transgenic plants

**FIGURE 10:**
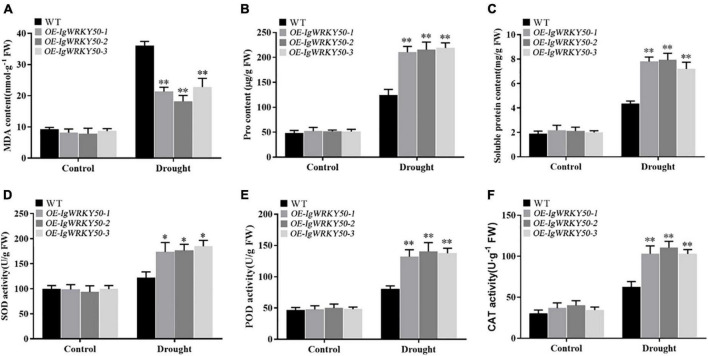
Changes of physiological indexes in WT and *IgWRKY50* transgenic lines under drought stress. **(A–F)** Are the changes of MDA content, Pro content, Soluble Protein content, SOD activity, POD activity, and CAT activity of WT and *IgWRKY50* transgenic lines under drought stress in sequence. Data are means ± *SD* of three independent experiments, and asterisks (*or**) represent the significant differences at *p* < 0.05 or *p* < 0.01, respectively (Student’s *t*-test).

**FIGURE 11:**
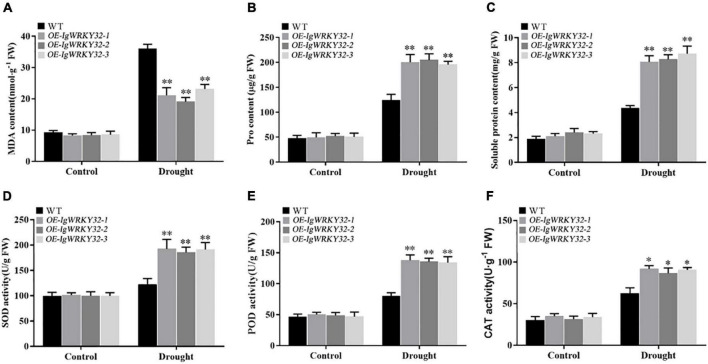
Changes of physiological indexes in WT and *IgWRKY32* transgenic lines under drought stress. **(A–F)** Are the changes of MDA content, Pro content, Soluble Protein content, SOD activity, POD activity, and CAT activity of WT and *IgWRKY32* transgenic lines under drought stress in sequence. Data are means ± *SD* of three independent experiments, and asterisks (*or**) represent the significant differences at *p* < 0.05 or *p* < 0.01, respectively (Student’s *t*-test).

To further confirm the role of *IgWRKY50* and *IgWRKY32* genes in the process of stress resistance of *Arabidopsis*, the physiological parameters of the WT and transgenic plants under drought stress for 14 days and normal water conditions were analyzed. Under normal conditions, the SOD, POD, and CAT activities and the MDA, Pro, SP contents were not significantly different between the WT and *IgWRKY50* and *IgWRKY32* transgenic lines. Under drought conditions, compared with the WT, the transgenic lines of *IgWRKY50* and *IgWRKY32* had a lower MDA content, a higher Pro and SP content, and higher SOD, POD, and CAT activity, but there was no significant difference between the *IgWRKY50* and *IgWRKY32* transgenic lines (Figures 10, 11). These results suggest that *IgWRKY50* and *IgWRKY32* transgenic plants may improve drought resistance by accumulating osmotic substances and regulating the reactive oxygen species (ROS) system.

### *IgWRKY50* and *IgWRKY32* changed the transcripts of the stress-responsive genes

**FIGURE 12:**
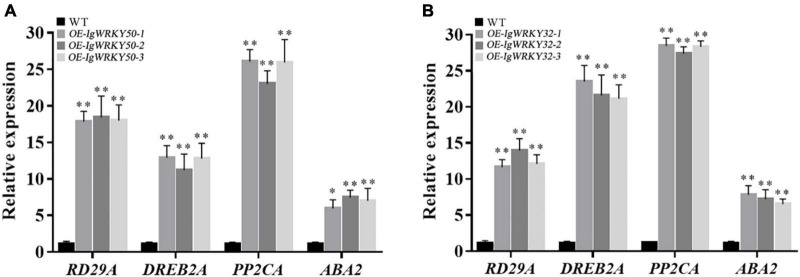
Expression levels of stress-responsive genes under regulation of *IgWRKY50* **(A)** and *IgWRKY32* **(B)**. The vertical ordinates are fold changes, and the horizontal coordinates are gene names. Data are means ± *SD* of three independent experiments, and asterisks (*or**) represent the significant differences at *p* < 0.05 or *p* < 0.01, respectively (Student’s *t*-test).

To explore the possible molecular mechanisms of *IgWRKY50* and *IgWRKY32* in stress responses, the relative expression levels of the stress-responsive genes were determined in transgenic and WT plants under drought conditions. The results showed that overexpression of *IgWRKY50* and *IgWRKY32* could promote the transcription of *RD29A, DREB2A, PP2CA*, and *ABA2*, especially the transcription of *PP2CA*, which reached a very high level in all transgenic lines. These results suggested that *IgWRKY50* and *IgWRKY32* may play a role in the drought stress response by regulating stress-related genes (Figure 12).

## Discussion

The WRKY transcription factor family, as one of the largest transcription factor families in plants, could widely participate in the defense regulatory network and play an important regulatory role in the response of plants to abiotic stresses (Chen et al., 2012). In the past few years, the functions of WRKY transcription factors have been extensively explored in many plants, such as soybean (Wu et al., 2017; Shi et al., 2018), cotton (Yu et al., 2012; Wang et al., 2019), maize (Cai et al., 2014; Hu et al., 2021), and wheat (Okay et al., 2014; Ye et al., 2021). However, there are few studies of garden plants, and the role of the *Iris germanica* WRKY transcription factor in mediating abiotic stress responses has not been investigated.

In this study, two new WRKY genes, *IgWRKY50* and *IgWRKY32*, were isolated from the *Iris germanica* cultivar “Little Dream,” and they belong to the WRKY transcription factor family Group II and Group III, respectively. Phylogenetic analysis showed that *IgWRKY50* and *IgWRKY32* had high homology with *Apostasia shenzhenica* and *Asparagus officinalis*, which belong to Liliidae. The IgWRKY50-GFP and IgWRKY32-GFP fusion proteins were exclusively localized to the nucleus of *Arabidopsis* protoplasts in a transient expression assay. This result was in agreement with their putative role as a transcription factor and similar to previous reports on some other WRKY TFs (Guo et al., 2011; Zhu et al., 2019). Most of the WRKY genes belonging to Group II and Group III could respond to drought stress signals. For example, the overexpression of wheat *TaWRKY1* and *TaWRKY33* genes (members of Group II and Group III) improved the drought resistance of *Arabidopsis* (He et al., 2016). *WRKY50* and *WRKY32* genes have been proven to be involved in plant hormone regulation and signal transduction pathways in *Arabidopsis* (Huang, 2018) and tobacco (Guo et al., 2018) under abiotic stress. At the same time, the significant expression patterns of *IgWRKY50* and *IgWRKY32* under PEG-6000, ABA and high-temperature stress also suggested that the two genes may play an important role in regulating the response of *Iris germanica* to abiotic stress.

WRKY gene overexpression could improve the tolerance of plants to multiple or single abiotic stresses (Jiang et al., 2012; Chu et al., 2015; Zhou et al., 2015). In our study, *IgWRKY50* and *IgWRKY32* were overexpressed in *Arabidopsis*, and the transgenic plants obtained a higher germination rate and a longer taproot length under osmotic stress. Transgenic *Arabidopsis* showed obvious drought resistance compared with WT under continuous natural drought. The water loss rate of the detached leaves has been widely used to reflect drought tolerance in plants (Li et al., 2020). The water loss rates of *IgWRKY50-* and *IgWRKY32-*overexpressing *Arabidopsis* plant isolated leaves were significantly lower than those of WT. This was consistent with the result that the stomatal closure of transgenic plants was higher than that of WT under PEG-6000 simulated drought stress. This result indicated that the overexpression of *IgWRKY50* and *IgWRKY32* improved the drought resistance of the transgenic plants to a certain extent.

Plants usually regulate their metabolism through a series of physiological and biochemical activities to reduce the damage caused by stress (Anjum et al., 2011). Drought stress often causes excessive accumulation of ROS and results in oxidative damage to cell components (Verslues et al., 2006). SOD, POD, and CAT are important antioxidant enzymes and they play an important role in scavenging active oxygen free radicals in plants (Wu et al., 2013). Many studies have shown that ROS scavenging ability was associated with plant tolerance to abiotic stress (Fang et al., 2015; Swain et al., 2017; Wang et al., 2017). Malondialdehyde (MDA) is one of the most important products of membrane lipid peroxidation (Grotto et al., 2009). Proline (Pro) and soluble proteins (SPs) play osmoregulatory roles in plant cytoplasm (Hanif et al., 2021). MDA, Pro, and SPs are the main indicators to determine plant stress resistance (Grotto et al., 2009; Jiao et al., 2012). Previous studies have shown that overexpression of *CKWRKY33* (isolated from *Caragana korshinskii*) in *Arabidopsis* could increase the activity of antioxidant enzymes and the content of osmotic regulators, reduce the accumulation of ROS and MDA, and thus improve the resistance of transgenic plants (Li et al., 2021). In this study, the SOD, POD and CAT activities and the proline and soluble protein contents of transgenic plants were significantly higher than those of the WT, while the MDA content was significantly lower than that of the WT under drought conditions, indicating that the *IgWRKY50* and *IgWRKY32* genes improve the drought resistance of plants by increasing the activities of antioxidant enzymes and accumulating more osmotic substances.

The ABA pathway plays a key role in regulating plant growth and development and in responding to abiotic stresses (Tuteja, 2007; Skubacz et al., 2016). When plants are in an adverse environment, they often respond to abiotic stress through ABA-dependent or ABA-independent pathways (Shinozaki and Yamaguchi-Shinozaki, 2007; Zhang et al., 2012). *PP2CA* (Ma et al., 2009; Nakashima et al., 2014), *ABA2* (Leoveanu, 2015; Nègre et al., 2019), *DREB2A* (Sakuma et al., 2006; Soma et al., 2021), and *RD29A* (Narusaka et al., 2003) are well known ABA responsive genes. The expression of *PP2CA* and *ABA2* (ABA-dependent) and *DREB2A* and *RD29A* (ABA-independent) genes were enhanced in I*gWRKY50*- and *IgWRKY32*-overexpressing transgenic *Arabidopsis thaliana* plants. It suggested that *IgWRKY50* and *IgWRKY32* may be involved in the regulation of drought stress through ABA-dependent and ABA-independent pathways. The similar findings were also found in the functions of *TaWRKY46* and *TaWRKY93* transcription factors. The former regulated *ABF3*, *RD29B*, *DREB2A*, *CBF2*, and *CBF3*, the latter acted on *ABF3*, *ABI1*, *ABI2*, *DREB2A*, *RD19A*, and *RD21* (Qin et al., 2015; Li et al., 2020). This may be because a single WRKT TF can participate in regulating several seemingly disparate processes (Rushton et al., 2010). The result of high expression of *PP2CA*, *ABA2, DREB2A*, and *RD29A* genes in transgenic *Arabidopsis thaliana* after drought stress is in accordance with previous findings that plants respond to abiotic stresses by enhancing the expression of stress-related genes (Sakuma et al., 2006; Bihmidine et al., 2013; He et al., 2016; Mao et al., 2016). It is speculated that the massive transcription of these genes involved in ABA signal transduction, thus the drought resistance of the transgenic lines is enhanced.

**FIGURE 13:**
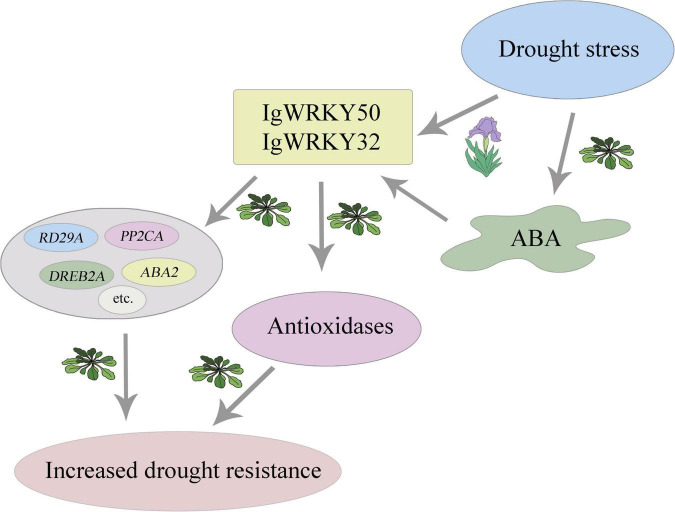
*IgWRKY50* and *IgWRKY32* of *Iris germanica* improve plant drought resistance by mediating ABA signaling pathway. Simulating drought stress with 200 g⋅L^–1^ PEG 6000 induced the expression of *IgWRKY50* and *IgWRKY32* in *Iris germanica. IgWRKY50* and *IgWRKY32* improved the drought resistance of transgenic *Arabidopsis* by up-regulating the expression of related genes in ABA-dependent and –independent pathways, increasing the activity of antioxidant enzymes, and reducing oxidative damage.

Based on the results of this study, we propose a diagram showing the role of *IgWRKY50* and *IgWRKY32* in the drought stress response regulation mechanism (Figure 13). Although *IgWRKY50* and *IgWRKY32* can improve the drought resistance of *transgenic Arabidopsis*, how *IgWRKY50* and *IgWRKY32* promote the expression of drought-responsive genes, how their products affect the network mechanism of drought resistance-related genes in *Arabidopsis* and how *IgWRKY50* and *IgWRKY32* function in *Iris germanica* plants still needs further verification.

## Conclusion

Two *Iris germanica* WRKY genes, *IgWRKY50* and *IgWRKY32*, were isolated and identified for the first time, and their characteristics were investigated in this study. The proteins of the two genes were located in the nucleus. The expression of *IgWRKY50* and *IgWRKY32* was induced by PEG-6000, high temperature and ABA stresses. Further research revealed that overexpression of *IgWRKY50* and *IgWRKY32* could improve the drought resistance of transgenic *Arabidopsis*, and the transgenic plants could accumulate more osmotic regulatory substances, reduced MDA content, and enhanced the activities of SOD, POD, and CAT under natural drought treatment. At the same time, *IgWRKY50* and *IgWRKY32* also activated the expression of stress response genes such as *RD29A, DREB2A, PP2CA*, and *ABA2*. The results of this study indicated that *IgWRKY50* and *IgWRKY32*, as positive regulators of drought stress, have potential applications in molecular breeding for drought resistance in *Iris*.

## Data availability statement

The datasets presented in this study can be found in online repositories. The names of the repository/repositories and accession number(s) can be found in the article/Supplementary material.

## Author contributions

DH and JZ conceived and designed this study. JZ, MZ, QW, XH, DD, BS, SW, and PS performed the research and collected data. JZ analyzed the data and wrote the manuscript. JZ and XZ revised the manuscript. All authors read and approved the final manuscript, contributed to the article, and approved the submitted version.

## Footnotes

1
https://www.ncbi.nlm.nih.gov/orffinder

2
http://smart.embl-heidelberg.de/

3
https://zhanglab.ccmb.med.umich.edu/I-TASSER/

4
https://www.rbvi.ucsf.edu/chimera

5
http://rsbweb.nih.gov/ij
