# Supplementary material for: Drought-responsive WRKY transcription factor genes IgWRKY50 and IgWRKY32 from Iris germanica enhance drought resistance in transgenic Arabidopsis
Source: Front Plant Sci. 2022 Sep 6;13:983600. doi: 10.3389/fpls.2022.983600 (PMC9486095; doi:10.3389/fpls.2022.983600)
Supplement: Supplementary file 1 [file Table_1.docx]

**Supplementary Table S1A.** List of primer sequences used for RT-qPCR of IgWRKY50 and IgWRKY32.

| Name of the primer | Primer sequence |
| --- | --- |
| *IgWRKY50*-qPCR-F | ATCAGTCAAGAACAGCCCCA |
| *IgWRKY50*-qPCR-R | GGCGTCCTGAGTAGCATAGT |
| *IgWRKY32*-qPCR-F | TTTGAAGTCGGCATCTCCCT |
| *IgWRKY32*-qPCR-R | CCTCTCGGATTCTCGTCTCC |
| *Actin11*-F | AAGA TTGATGAGAAGCGAAAGG |
| *Actin11*-R | GTTTTCAGGAAGAGCAGCCA |

**Supplementary Table S1B.** List of primer sequences used for amplification of IgWRKY50 and IgWRKY32.

| Name of the primer | Primer sequence |
| --- | --- |
| IgWRKY50-F1 | CCTCTTCCTCCTCCATCGTC |
| IgWRKY50-R1 | GCCAAGCCCACCAACATTTA |
| IgWRKY50-F2 | ATGATGGCCGATGACTTCCTC |
| IgWRKY50-R2 | TCAAGTCGATGAATTAGGAG |
| IgWRKY32-F1 | GCCATGGATCCAGAAAGCAC |
| IgWRKY32-R1 | AGACTTCGCCTAGCTTTGGT |
| IgWRKY32-F2 | ATGGATCCAGAAAGCACTCG |
| IgWRKY32-R2 | TTATAGGTAGGCTGATGCATC |
