# Supplementary material for: Drought-responsive WRKY transcription factor genes IgWRKY50 and IgWRKY32 from Iris germanica enhance drought resistance in transgenic Arabidopsis
Source: Front Plant Sci. 2022 Sep 6;13:983600. doi: 10.3389/fpls.2022.983600 (PMC9486095; doi:10.3389/fpls.2022.983600)
Supplement: Supplementary file 2 [file Table_2.DOCX]

**Supplementary Table S2.** WRKY transcription factor family genes for phylogenetic analysis

| Gene name | Accession number | Species |
| --- | --- | --- |
| *AcWRKY50* | XP_020098275.1 | *Ananas comosus* |
| *AcWRKY51* | PSS30261.1 | *Actinidia chinensis* var*. chinensis* |
| *AcWRKY51-2* | PSS30471.1 | *Actinidia chinensis* var. *chinensis* |
| *AcWRKY51-3* | OAY77747.1 | *Ananas comosus* |
| *AcWRKY70* | PSS34769.1 | *Actinidia chinensis* var*. chinensis* |
| *AoWRKY32* | XP_020244424.1 | *Asparagus officinalis* |
| *AoWRKY34* | XP_020244425.1 | *Asparagus officinalis* |
| *AoWRKY46* | XP_020244498.1 | *Asparagus officinalis* |
| *AoWRKY46-2* | XP_020241688.1 | *Asparagus officinalis* |
| *AoWRKY50* | XP_020242404.1 | *Asparagus officinalis* |
| *AsWRKY51* | PKA57309.1 | *Apostasia shenzhenica* |
| *AtWRKY51* | XP_020192531.1 | *Aegilops tauschii* subsp. *tauschii* |
| *AtWRKY57* | XP_020147509.1 | *Aegilops tauschii* subsp. *tauschii* |
| *AtWRKY57-2* | XP_020195305.1 | *Aegilops tauschii* subsp. *tauschii* |
| *AtWRKY59* | XP_020195304.1 | *Aegilops tauschii* subsp. *tauschii* |
| *BdWRKY48* | XP_003568011.1 | *Brachypodium distachyon* |
| *BdWRKY62* | XP_003569675.1 | *Brachypodium distachyon* |
| *CmWRKY51* | XP_008457906.1 | *Cucumis melo* |
| *CsWRKY70* | XP_028055071.1 | *Camellia sinensis* |
| *CsWRKY70-2* | XP_028094837.1 | *Camellia sinensis* |
| *DcWRKY57* | XP_020696170.1 | *Dendrobium catenatum* |
| *DoWRKY50* | OEL32659.1 | *Dichanthelium oligosanthes* |
| *EgWRKY50* | XP_010935437.1 | *Elaeis guineensis* |
| *EgWRKY51* | XP_010925052.1 | *Elaeis guineensis* |
| *EgWRKY51-2* | XP_010915169.1 | *Elaeis guineensis* |
| *EgWRKY51-3* | XP_010925387.1 | *Elaeis guineensis* |
| *EgWRKY51-4* | XP_010925386.1 | *Elaeis guineensis* |
| *EgWRKY51-5* | XP_018717773.1 | *Eucalyptus grandis* |
| *EgWRKY51-6* | XP_010027762.1 | *Eucalyptus grandis* |
| *EgWRKY70* | XP_019706198.1 | *Elaeis guineensis* |
| *EgWRKY70-2* | XP_010931380.1 | *Elaeis guineensis* |
| *EgWRKY70-3* | XP_010921157.1 | *Elaeis guineensis* |
| *GmWRKY50* | XP_003522275.1 | *Glycine max* |
| *GsWRKY50* | XP_028227831.1 | *Glycine soja* |
| *HvWRKY19* | AGM37863.1 | *Hordeum vulgare* subsp. *vulgare* |
| *HvWRKY19-2* | KAE8773823.1 | *Hordeum vulgare* |
| *HvWRKY20* | ABI13386.1 | *Hordeum vulgare* |
| *LsWRKY70* | XP_023772872.1 | *Lactuca sativa* |
| *MaWRKY50* | XP_009393635.2 | *Musa acuminata* subsp. *malaccensis* |
| Gene name | Accession number | Species |
| *McWRKY51* | XP_022157717.1 | *Momordica charantia* |
| *MdWRKY51* | XP_008342835.2 | *Malus domestica* |
| *NnWRKY51* | XP_019053770.1 | *Nelumbo nucifera* |
| *NnWRKY70* | XP_010252044.1 | *Nelumbo nucifera* |
| *NnWRKY70-2* | XP_010253044.1 | *Nelumbo nucifera* |
| *ObWRKY26* | XP_006654710.1 | *Oryza brachyantha* |
| *ObWRKY50* | XP_015689727.1 | *Oryza brachyantha* |
| *OsWRKY24* | XP_015635650.1 | *Oryza sativa Japonica Group* |
| *OsWRKY50* | XP_015637886.1 | *Oryza sativa Japonica Group* |
| *OsWRKY59* | KAB8083203.1 | *Oryza sativa* |
| *PaWRKY50* | XP_021824155.1 | *Prunus avium* |
| *PdWRKY50* | XP_008810071.1 | *Phoenix dactylifera* |
| *PdWRKY50-2* | XP_008812294.1 | *Phoenix dactylifera* |
| *PdWRKY51* | XP_008793280.1 | *Phoenix dactylifera* |
| *PdWRKY70* | XP_017697073.1 | *Phoenix dactylifera* |
| *PdWRKY70-2* | XP_008788524.1 | *Phoenix dactylifera* |
| *PeWRKY51* | XP_020591477.1 | *Phalaenopsis equestris* |
| *PeWRKY51-2* | XP_020591476.1 | *Phalaenopsis equestris* |
| *PhWRKY57* | XP_025817958.1 | *Panicum hallii* |
| *PmWRKY50* | XP_008220527.1 | *Prunus mume* |
| *PmWRKY51* | XP_016649132.1 | *Prunus mume* |
| *PmWRKY51-2* | XP_008232443.1 | *Prunus mume* |
| *PpWRKY50* | XP_020409575.1 | *Prunus persica* |
| *PpWRKY51* | XP_007206237.1 | *Prunus persica* |
| *PyWRKY51* | PQP97094.1 | *Prunus yedoensis* var*. nudiflora* |
| *QsWRKY70* | XP_023923322.1 | *Quercus suber* |
| *SbWRKY47* | XP_002450147.1 | *Sorghum bicolor* |
| *SbWRKY50* | XP_002440147.1 | *Sorghum bicolor* |
| *SbWRKY50-2* | XP_002455037.1 | *Sorghum bicolor* |
| *SiWRKY50* | XP_012700101.1 | *Setaria italica* |
| *SiWRKY57* | XP_004969741.1 | *Setaria italica* |
| *TcWRKY70* | XP_017975966.1 | *Theobroma cacao* |
| *TuWRKY50* | EMS51319.1 | *Triticum urartu* |
| *TuWRKY51* | EMS46328.1 | *Triticum urartu* |
| *VuWRKY70* | XP_027926250.1 | *Vigna unguiculata* |
| *VvWRKY70* | XP_002272504.1 | *Vitis vinifera* |
| *ZmWRKY50* | NP_001338874.1 | *Zea mays* |
| *ZmWRKY50-2* | XP_008675106.1 | *Zea mays* |
| *ZmWRKY50-3* | ONM38747.1 | *Zea mays* |
| *ZmWRKY50-4* | PWZ16237.1 | *Zea mays* |
| *ZmWRKY51* | PWZ16729.1 | *Zea mays* |
| *ZmWRKY51-2* | XP_008675106.1 | *Zea mays* |
