# Supplementary material for: Drought-responsive WRKY transcription factor genes IgWRKY50 and IgWRKY32 from Iris germanica enhance drought resistance in transgenic Arabidopsis
Source: Front Plant Sci. 2022 Sep 6;13:983600. doi: 10.3389/fpls.2022.983600 (PMC9486095; doi:10.3389/fpls.2022.983600)
Supplement: Supplementary file 3 [file Table_3.docx]

**Supplementary Table S3A.** List of primer sequences used to construct of PBI221-*IgWRKY50*-GFP and PBI221-*IgWRKY32*-GFP.

| Name of the primer | | | Primer sequence |
| --- | --- | --- | --- |
| PBI221-*IgWRKY50*-GFP -F | | | TTCATTTGGAGAGAACACGGGGGACTCTAG  AATGATGGCTGACGACTTCTTGCCA |
| PBI221-*IgWRKY50*-GFP -R | | | CCTCGCCCTTGCTCACCATAAGCTTGTCGACTGTAGAAG  AGTTAGGAGGTGTAGTTGTA |
| PBI221-*IgWRKY32*-GFP-R | TTCATTTGGAGAGAACACGGGGGACTCTAGAA  TGGATCCAGAGTCTACAAGGATCGAC | | |
| PBI221-*IgWRKY32*-GFP-R | | CCTCGCCCTTGCTCACCATAAGCTTGTCGACCAAGTAAGCT  GATGCATCTGGTCCGTAGAACTG | |

**Supplementary Table S3B.** List of primer sequences used to construct of PBI121-*IgWRKY50* and PBI121-*IgWRKY32*.

| Name of the primer | Primer sequence |
| --- | --- |
| PBI121-*IgWRKY50* -F | GACACTCTAGAATGATGGCTGATGATTTTCT |
| PBI121-*IgWRKY50* -R | GTGTCCCCGGGCTAGTAGAACTATTAG |
| PBI121-*IgWRKY32* -F | GACACTCTAGAATGGATCCTGAATCTACTA |
| PBI121-*IgWRKY32* -R | GTGTCCCCGGGCTAAGGTAAGCAGAAGCATCAGG |

**Supplementary Table S3C.** List of primer sequences used for RT-qPCR of Stress-Related Genes.

| Name of the primer | Primer sequence |
| --- | --- |
| *ABA2-F* | ATTGATCACTGGAGGAGCCACAG |
| *ABA2-R* | ATTACGAATATCAGGGCACGGTG |
| *PP2CA -F* | ATGTCAGAGACCAACAAGAATGCC |
| *PP2CA -R* | CTACTTGTTCAGGCCGGTCTTG |
| *RD29A-F* | ATCACTTGGCTCCACTGTTGTTC |
| *RD29A-R* | ACAAAACACACATAAACATCCAAAGT |
| *DREB2A-F* | GACCTAAATGGCGACGATGT |
| *DREB2A-R* | TCGAGCTGAAACGGAGGTAT |
| *Actin8-F* | CTCAGGTATTGCAGACCGTATGAG |
| *Actic8 -R* | CTGGACCTGCTTCATCATACTCTG |
