# Supplementary material for: Drought-responsive WRKY transcription factor genes IgWRKY50 and IgWRKY32 from Iris germanica enhance drought resistance in transgenic Arabidopsis
Source: Front Plant Sci. 2022 Sep 6;13:983600. doi: 10.3389/fpls.2022.983600 (PMC9486095; doi:10.3389/fpls.2022.983600)
Supplement: Supplementary file 4 [file Table_4.docx]

**Supplementary Table S4A.** BLAST comparison result of *IgWRKY50* amino acid sequence

| Species | Total | E value | Ident | Accession |
| --- | --- | --- | --- | --- |
| *Musa acuminata* subsp.*malaccensis* | 263 | 1.00E-65 | 82.56% | XM_009395360.2 |
| *Zea mays* | 255 | 2.00E-63 | 86.73% | NM_001329795.1 |
| *Panicum hallii* | 244 | 4.00E-60 | 84.55% | XM_008795058.3 |
| *Sorghum bicolor* | 242 | 5.00E-59 | 85.92% | XM_002440682.2 |
| *Elaeis guineensis* | 235 | 2.00E-57 | 78.91% | XM_010937135.3 |
| *Ananas comosus* | 234 | 7.00E-57 | 80.16% | XM_020242686.1 |
| *Oryza sativa japonica* | 233 | 2.00E-56 | 84.36% | XM_015782532.2 |
| *Setaria Italica* | 218 | 5.00E-52 | 83.33% | XM_004968427.3 |
| *Aegilops tauschii* subsp. *Tauschii* | 214 | 7.00E-51 | 83.58% | XM_020314057.1 |
| *Phoenix dactylifera* | 214 | 7.00E-51 | 81.74% | XM_008810692.1 |
| *Juglans regia* | 207 | 1.00E-48 | 82.46% | XM_018955219.1 |
| *Asparagus officinalis* | 204 | 1.00E-47 | 76.36% | XM_020386815.1 |
| *Eucalyptus grandis* | 201 | 4.00E-47 | 81.40% | XM_018862228.1 |
| *Prunus mume* | 199 | 1.00E-46 | 80.95% | XM_008795059.3 |
| *Syzygium oleosum* | 198 | 5.00E-46 | 77.38% | XM_012844647.3 |

**Supplementary Table S4B.** BLAST comparison result of *IgWRKY32* amino acid sequence

| Species | Total | E value | Ident | Accession |
| --- | --- | --- | --- | --- |
| *Asparagus officinalis* | 109 | 3.00E-19 | 76.62% | XM_020388835.1 |
| *Durio zibethinus* | 69.8 | 2.00E-07 | 82.35% | XM_022905205.1 |
| *Abrus precatorius* | 65.3 | 1.00E-05 | 74.29% | XM_027478110.1 |
| *Rhodamnia argentea* | 69.8 | 2.00E-07 | 82.35% | XM_030684521.1 |
| *Amborella trichopoda* | 69.8 | 2.00E-07 | 76.77% | XM_006836704.3 |
| *Phoenix dactyllifera* | 69.8 | 2.00E-07 | 81.25% | XM_008784445.2 |
| *Punica granatum* | 65.3 | 1.00E-05 | 80.88% | XM_031515584.1 |
| *Syzygium oleosum* | 65.3 | 1.00E-05 | 80.88% | XM_030612717.1 |
| *Juglans regia* | 65.3 | 1.00E-05 | 80.88% | XM_018963740.1 |
| *Eucalyptus grandis* | 65.3 | 1.00E-05 | 80.88% | XM_010032770.2 |
| *Vitis vinifera* | 64.4 | 1.00E-05 | 81.54% | XM_002275365.4 |
| *Quercus suber* | 63.5 | 3.00E-05 | 75.86% | XM_024042425.1 |
| *Spinacia oleracea* | 63.5 | 3.00E-05 | 75.86% | XM_021993347.1 |
| *Vigna unguiculata* | 60.8 | 1.00E-04 | 76.92% | XM_028061783.1 |
| *Glycine soja* | 59 | 4.00E-04 | 76.62% | XM_028376622.1 |
| *Papaver somniferum* | 59 | 4.00E-04 | 75.61% | XM_026529656.1 |
| *Ricinus communis* | 59 | 4.00E-04 | 75.53% | XM_015725308.2 |
| *Hevea brasiliensis* | 59 | 4.00E-04 | 74.47% | XM_021825238.1 |
| *Glycine max* | 59 | 4.00E-04 | 76.62% | XM_003524062.3 |
