# Supplementary figures and images for: Drought-responsive WRKY transcription factor genes IgWRKY50 and IgWRKY32 from Iris germanica enhance drought resistance in transgenic Arabidopsis
Source: Front Plant Sci. 2022 Sep 6;13:983600. doi: 10.3389/fpls.2022.983600 (PMC9486095; doi:10.3389/fpls.2022.983600)

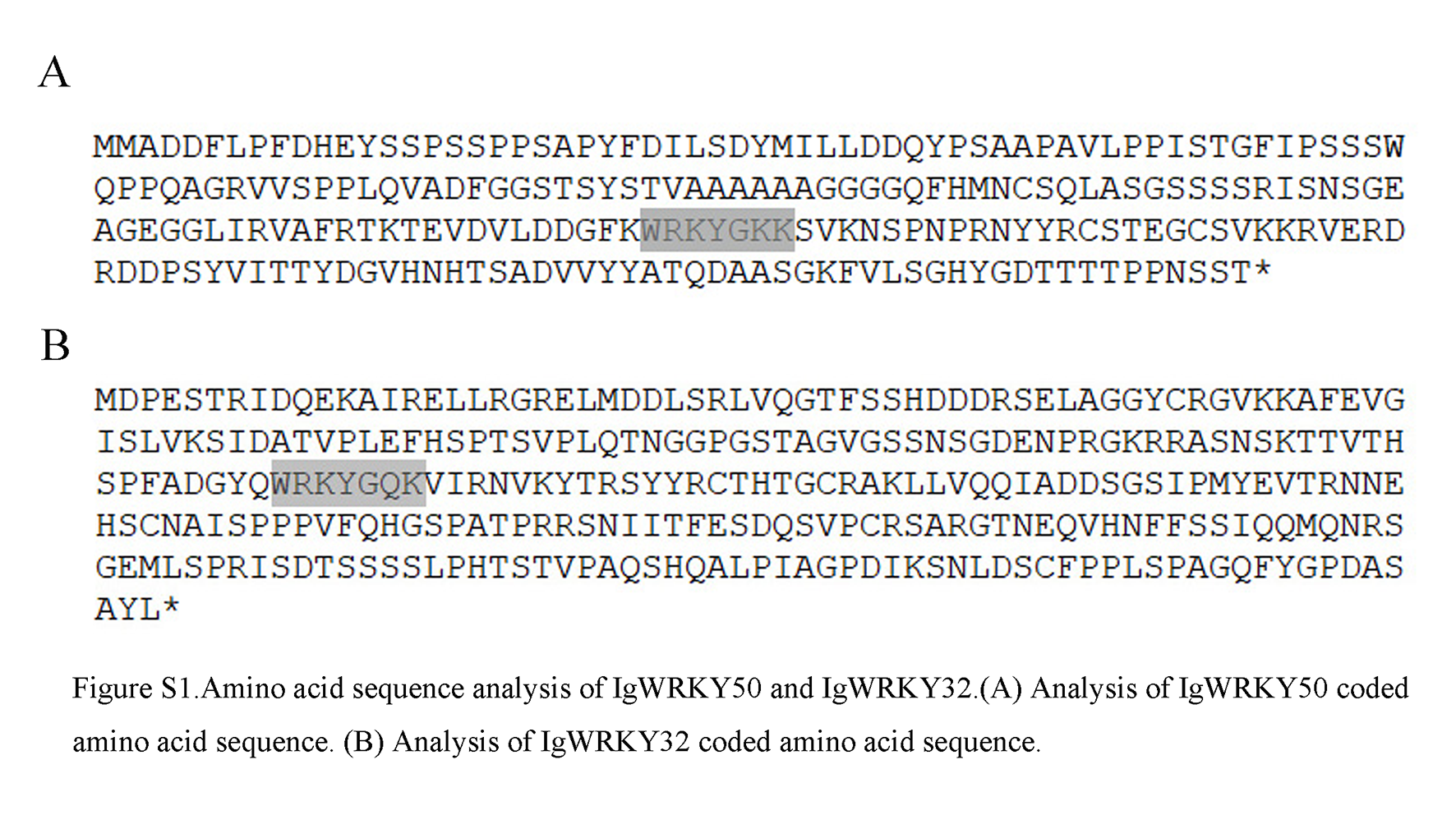

Supplement: Supplementary file 5 [file Image_1.TIF]
